# A Survey on Socially Assistive Robotics: Clinicians’ and Patients’ Perception of a Social Robot within Gait Rehabilitation Therapies

**DOI:** 10.3390/brainsci11060738

**Published:** 2021-06-02

**Authors:** Denniss Raigoso, Nathalia Céspedes, Carlos A. Cifuentes, Antonio J. del-Ama, Marcela Múnera

**Affiliations:** 1 Department of Biomedical Engineering, Colombian School of Engineering Julio Garavito, Bogota 111166, Colombia; denniss.raigoso@mail.escuelaing.edu.co (D.R.); marcela.munera@escuelaing.edu.co (M.M.); 2 Centre for Advanced Robotics at Queen Mary University of London, Mile End Rd, Bethnal Green, London E1 4NS, UK; n.cespedesgomez@qmul.ac.uk; 3 Electronic Technology department, Rey Juan Carlos University, 28933 Móstoles, Spain; antonio.delama@urjc.es; 4 Biomechanics and Assistive Technology Unit, National Hospital for Paraplegics, 45071 Toledo, Spain

**Keywords:** social robots, physical rehabilitation, perception, gait rehabilitation, lokomat

## Abstract

A growing interest in Socially Assistive Robotics in Physical Rehabilitation is currently observed; some of the benefits highlight the capability of a social robot to support and assist rehabilitation procedures. This paper presents a perception study that aimed to evaluate clinicians’ and patients’ perception of a social robot that will be integrated as part of Lokomat therapy. A total of 88 participants were surveyed, employing an online questionnaire based on the Unified Theory of Acceptance and Use of Technology (UTAUT). The participants belong to two health care institutions located in different countries (Colombia and Spain). The results showed an overall positive perception of the social robot (>60% of participants have a positive acceptance). Furthermore, a difference depending on the nature of the user (clinician vs. patient) was found.

## 1. Introduction

According to the World Health Organization (WHO), 15% of the world population has some type of disability. These disabilities are mainly caused by pathologies associated with strokes (CVA) and spinal injuries that lead to deficiencies in the motor functions. As a consequence, people’s independence was reduced, and their inclusion in society is affected [1]. In this context, Physical Rehabilitation (PR) is one of the strategies that is mainly used to improve the quality of life of this population by helping the users to increase their physical capabilities [2]. PR has three main aims: (i) to offer continuous monitoring considering the objectives established at the beginning of the rehabilitation; (ii) to promote and maintain the optimal sensory, intellectual, psychological and social level of the patient; and (iii) integrating the patient into society [2].

**Figure 1:**
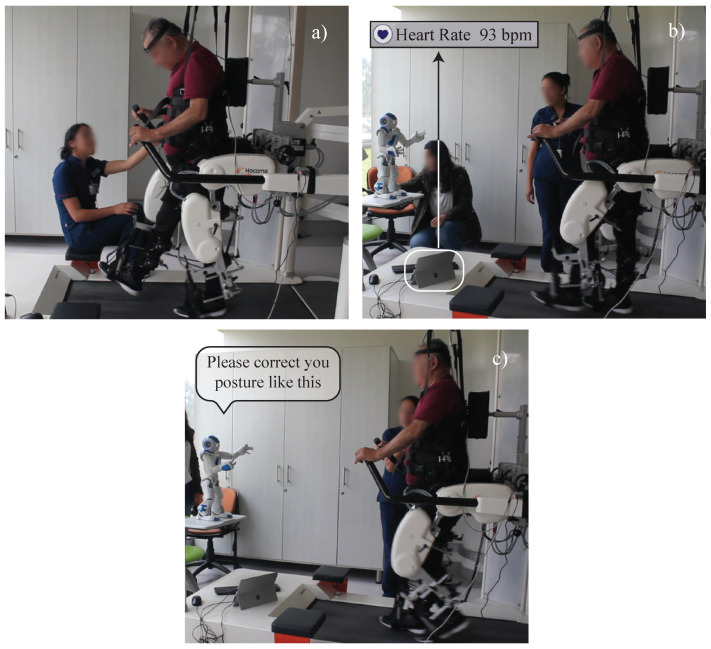
Video scenes of the robot assistance during Lokomat Rehabilitation. (**a**) Clinicians tasks performed during the conventional therapy, (**b**) On-line robot monitoring (e.g., heart rate acquisition), and (**c**) Robot supporting and providing feedback to the patient.

Within the PR, several technologies have been developed and implemented. For instance Walkbot (P&S Mechanics, Korea), Prodrobot (Prodromus, Germany) and Lokomat (Hocoma, Switzerland), Lokomat is one of the most used tools in this area [3], This technology supports gait rehabilitation by providing body weight support (BWS) and treadmill exercise. Lokomat is a robotic orthotic device that adjusts to the patient’s lower limbs with the general purpose of retraining the gait through repetitive and intensive simulation exercises (Figure 1a). It uses sensory stimulation fed by proprioceptive feedback games, improving the neuroplasticity for functional recovery. Several studies have demonstrated the efficacy of Lokomat in improving the motor control of the body [4], the muscle mass regaining [5], the gait speed, and the muscle capabilities [6]. Furthermore, Lokomat provides objective assessments by using quantitative indicators offering the clinicians a complete perspective of the patient’s physiological progress [7].

Despite the benefits of the Lokomat therapies, two limitations need to be addressed: (i) the *multi-tasking processes* performed by the clinicians during the therapy; and (ii) the need of providing *patient’s cognitive support*. During previous observations performed by the research group, it was seen that the healthcare staff continuously gave feedback to the patients about several physiological parameters at the same time. These *multi-tasking processes* can affect the quality of the assistance provided by the clinicians towards the patients, since there are several variables that must be measured during therapy, such as heart rate, posture and space-time parameters of gait [8,9]. Moreover, PR has to be a comprehensive strategy that includes social and psychological approaches to increase patients’ engagement and motivation [10]. Motivation is a fundamental tool in establishing adherence to a therapy regimen or task scenario and promoting behavior change [11]. Intrinsic motivation provides stimulation that drives an individual to adopt or change behavior to obtain internal satisfaction or fulfillment [12]. The intrinsic motivation can be affected by external factors [11]. In this case, the robot can influence intrinsic motivation across positive feedbacks.

Socially Assistive Robotics (SAR) is the field dedicated to developing robots that provide feedback, instructions, encouragement, and emotional support through social interaction to increase patient’s motivation and performance within the therapies. SAR has been initially defined as assistive Robotics (AR) and Social Interactive Robotics (SIR). In the first place, SAR and AR are meant to assist human users. However, in SAR, this assistance is specifically achieved by employing social interaction with the user. From this perspective, SAR and SIR have the same goal. They are focused on developing social interaction strategies that enable them to exhibit a closer and more effective interaction with the human user. Unlike SIR, the scope of SAR is limited to achieve major progress in the areas such as rehabilitation, healthcare, and so forth [13].

The main role of social robotic agents or social robots is to act as companions or assistants in a specific task. In rehabilitation and healthcare environments, social robots are regarded as training assistants, coaches, or motivator agents that help improving patient’s performance or increasing engagement during the therapy. With this in consideration, social robots must contain a series of features that allow them to interact effectively, providing adaptability and flexibility to human environments. As these agents are designed to interact socially with humans, they must exhibit human-like behaviors. Social robots must structure their appearance and functionality so that humans can interpret and be familiarized with [13].

Based on this, to enhance and improve the rehabilitation procedures provided by the Lokomat, complementary tools such as Socially Assistive Robotics (SAR) can be implemented. SAR systems provide support to the clinicians taking measures of physiological variables in real-time. However social robots also assist users in cognitive ways promoting social skills and interaction [14]. Social interaction is essential in rehabilitation; environmental-factors can generate motivation in the patient to reach a goal during the rehabilitation therapy. In order to maintain the social interaction between the robots and the users, acceptance and perceptions are being evaluated [15].

SAR has been used in several areas (e.g., education [16], home-based applications [17], health-care [18] among others), showing positive results. Specifically, several researchers had observed the SAR effects in PR. In [19] and [20], a humanoid robot (NAO) was integrated into pediatric rehabilitation. The study explores the issues and effects when a social robot is included in rehabilitation sessions for children. The results suggested that the support given by the robot tends to increase children’s activity and engagement during the session. Moreover, social robots have been used within post-stroke rehabilitation [21], showing potential benefits for the patients (e.g., increased willingness to perform prescribed exercises and enthusiastic responses towards the robot). Within the research of our group, social robots were integrated into rehabilitation scenarios [13,22] to enhance conventional rehabilitation procedures and support the health care staff.

Casas et al. [10,23] present a perception study applied to the clinicians and patients who participate in the Cardiac Rehabilitation (CR) service. A questionnaire based on the unified theory of acceptance and the use of technology (UTAUT) was implemented. To achieve the goal of this study. The researchers compared the perception of patients who never used the robot and the patients who were assisted by the robot in CR sessions. A survey to evaluate the attitudes towards using a social robot was developed and evaluated [24]. The attitude towards using the social robot-Chinese version (ATTUSRC) questionnaire was applied to 416 health professionals [24]. Demonstrating that health personnel had a positive attitude towards the robot in a long-term application, they viewed the robot as beneficial and practical in psycho-social care for the elderly. In our previous study [25], assessing a social robot’s short-term effects in patients’ cervical posture was developed. The robot acted as a therapy assistant to give feedback and motivation. Preliminary observations showed positive effects regarding robot monitoring and improvement of the spine posture [25] Moreover, to understand the long-term effects, in [26] a clinical validation with ten patients during 15 sessions were conducted in a rehabilitation center located in Colombia. Outcomes showed that the robot’s support improves the patients’ physiological progress by reducing their unhealthy spinal posture time, with positive acceptance. Additionally, a short questionnaire was applied to measure the stakeholders perception, 65% of patients described the platform as helpful and secure; and the health care staff agreed (>95%) that this tool can promote physical distancing and it is advantageous to support neurorehabilitation throughout the pandemic.

Although an initial perception was measured in previous studies, assessing the acceptance in a comprehensive manner is essential to increase the adherence to new technologies. For instance, Broadbent et al. [27] remark that the users’ needs and concerns regarding social robots have to be considered to improve the long-term interaction between human and social robots. In [28], the authors recommend using perception observation to enhance the inclusion of technologies into rehabilitation scenarios. Besides, there is a lack of studies that measure the stakeholder’s perception towards social robots and their roles in PR. Therefore, this study seeks to know the perception of clinicians and patients when involving both technologies, the social robot NAO and Lokomat. The findings of this survey will provide meaningful information to deploy the NAO robot in a real scenario of neurorehabilitation. Based on this, this paper is organized as follows: Section 2 describes the assessment method used to evaluate the perception towards social robotics. Section 3 introduces the results of the perception of clinicians and patients regarding the implementation of social robotics of assistance in gait rehabilitation assisted by Lokomat in Colombia and Spain. Finally, the results and conclusions are presented at the end of this paper.

## 2. Materials and Methods

This section describes the methodology implemented within a perception study towards healthcare professionals and patients involved in PR with Lokomat. Three steps were performed in order to achieve the perception and acceptance assessment: (i) the *hypothesis formulation*, (ii) the *experimental protocol* executed during the study; and (iii) the *Data Analysis*.

### 2.1. Hypothesis Formulation

To evaluate the differences between the participants involved in the study (i.e., clinicians and patients), regarding the social robot role in PR, the following hypotheses are formulated:

**Hypothese 1 (H1)**. *The perception between the clinicians and the patients is not significantly different*.

**Hypothese 2 (H2)**. *The perception between the clinicians and patients is significantly different*.

The perceptions of clinicians and patients are compared to understand their interests as future users to promote their adherence to the robot. It will help to understand their needs and identify guidelines for future developments in this field, such as defining the robot behavior and role during the interaction with clinicians and patients and, defining the interface design according to the most important constructs for each group.

### 2.2. Experimental Protocol

An online questionnaire was applied to patients and healthcare personnel who have not been assisted or supported by a social robot during the rehabilitation processes. Before conducting the survey, the participants had to watch a video where the robot’s roles were elucidated. Thus, the understanding of the application can be more explicit. Patients included in the study do not present cognitive impairments that do not allow the understanding of the robot’s instructions and also people who have agreed to participate voluntarily in the study. There was no limit on the age of the participants, but if the respondent is under 18 years of age, the legal guardian completes the questionnaire. This study was approved by the ethics committee of the Colombian School of Engineering Julio Garavito.

The study was performed in three different rehabilitation centers. Two of these rehabilitation centers are located in Colombia, Bogotá (Mobility Group and Clínica Universidad de la Sabana), and a center located in Spain, Toledo (Hospital Nacional de Parapléjicos).

A total of 88 participants were recruited to complete the questionnaire online. Table 1 showed the details of the participants and their demographic data.

#### Procedure

As mentioned in previous sections, two main steps were performed: (i) the *Technology Explanation* and (ii) the *Questionnaire Implementation*. The *Technology Explanation* was performed to inform the patients and clinicians about the possible robot’s role during the rehabilitation procedure. Several studies [14,29], recommend this first step to achieve a better understanding of the technology dimensions (i.e., the robot’s limitations, capabilities, and tasks). In this context, the robot presented in the video is the NAO robot; this social robot has a humanoid appearance that allows performing specific animations and behaviors that are essential in the rehabilitation procedure (e.g., arms movement and body gestures) [30] and was introduced to the participants employing a video, the video was recorded during a pilot study with the social robot [25]. In the video, three robot tasks were highlighted: (i) clinicians within the therapy of Lokomat had to carry out multitasking processes to achieve complete patient performance. For instance, one of the most noticeable tasks is the correction of the patient’s thoracic and cervical posture in the coronal, axial and sagittal plane, while the physiotherapist corrects at the same time the ankle posture. (Figure 1a); (ii) patient’s online monitoring through a sensory interface capable of acquiring measurements of heart rate, posture, and spatiotemporal parameters of gait (Figure 1b); and (iii) provide verbal and non-verbal feedback provided by the robot (Figure 1c).

Finally, the *Questionnaire Implementation* aimed to evaluate the perception of the patients, using the Almere Model [31]. The Almere Model is adapted from the Unified Theory of Acceptance and Use of Technology (UTAUT) questionnaire [32]. This questionnaire is based on 40 questions (Appendix A, Table A1) aimed to assess the perception of the participants through different constructs (e.g., *Psychological factor* (PF), *Social perception* (SP), *Entertainment Level* (LR), *Effort’s Expectations* (EE), *Performance Expectations* (PE) and *Facility Conditions* (FC)). Table 2 present the definitions of each construct. The questions are divided into 36 closed questions. 32 items were evaluated through a 5-point Likert scale (i.e., 1: strongly disagree to 5: strongly agree), 4 dichotomous type questions answered with three scores (i.e., Yes, No, Maybe); and 4 open questions. In the case of the closed questions of the questionnaire, positive (e.g., “The therapy is more enjoyable if a robot participates in it”) and negative (e.g., “The therapy can be bored by using the robot”) the formulation was used to avoid the bias in the results.

### 2.3. Data Analysis

The analysis of the data was deployed in two parts: (i) to determine the general perspective of the population towards robotic assistance; and (ii) a comparative analysis using the Mann Withney Test was performed to determine the p-value of each of the constructs, aimed to compare the perception and significant differences between the two participant groups and also to evaluate the normality of the questions that are not normal (i.e., clinicians and patients). Additionally, to analyze the open questions (Appendix A, Table A1) textual data analysis was performed. The analysis was implemented in Excel and SPSS software.

## 3. Results

The results of 88 surveys are presented in this section. As it was mentioned the development of the questionnaires was carried out in Colombia and Spain.

### 3.1. Questionnaire Reliability

To measure the reliability of the UTAUT questionnaire applied in this study, the Cronbach’s method [33,34] was followed up. Cronbach’s alpha is computed by correlating the score for each scale item with the total score for each observation and then comparing that correlation to the variance for all individual item scores. Individual variance refers to the variance associated with each item, and total variance refers to the variance associated with the observed total scores. Equations (1) and (2) were used in the calculation. *K*: Represent the number of questions or items in the questionnaire, $S_{i}^{2}$: Individual variance (variance of item i), it is the variance obtained in each element, each value will be added to get $\Sigma_{i=1}^{K}S_{i}^{2}$ factor, $S_{t}^{2}$: Is the variance of the total observed values and *α:* Cronbach’s alpha coefficient.

(1) $$K=36\;\Sigma_{i=1}^{K}S_{i}^{2}=45.07\;S_{t}^{2}=286.41$$

(2) $$\begin{array}{c} \alpha =\frac{K}{K-1}*\left(1-\frac{\Sigma_{i=1}^{K}S_{i}^{2}}{S_{t}^{2}}\right)\\ \alpha =\frac{36}{35}*\left(1-\frac{45.07}{286.41}\right)\\ \alpha =0.86. \end{array}$$

As it can be seen, the Cronbach’s alpha coefficient shows that the questionnaire is reliable and can be applied during the development of this study.

### 3.2. Overall User’s Perception

**Figure 2:**
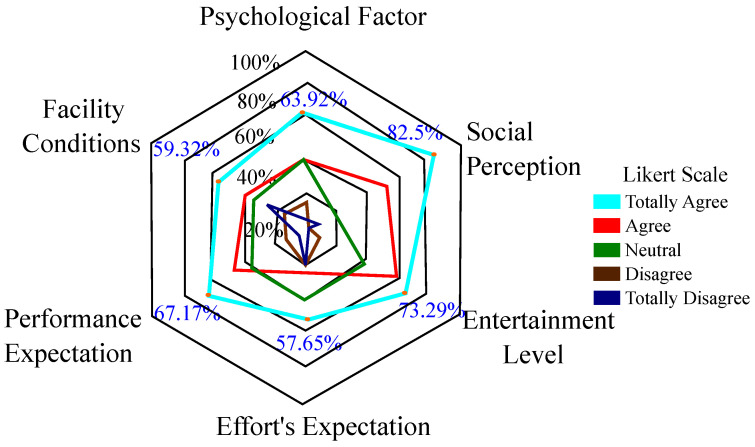
Overall perception (clinicians and patients) regarding the UTAUT Questionnaire results.

Figure 2 shows the overall perception between clinicians and patients regarding the questionnaire. As it can be seen, the perception towards the robot is mostly positive for the constructs proposed in the UTAUT questionnaire (PF, 63.92%; SP, 82.5%; EL, 73.29%, and PE, 67.17%). For example, in the case of the *Social Perception* and the *Entertainment Level* constructs, a greater number of participants have answered with a high score (i.e., “Agree” and Totally Agree”) showing an initial constructive perception. On the other hand, the negative perception (i.e., “Neutral”, “Disagree” and “Totally Disagree scores”) is lower, with a greater number of answers in the *Effort’s Expectation* and *Facility Conditions* constructs (EE, 51.14%; FE, 43.63%).

**Figure 3:**
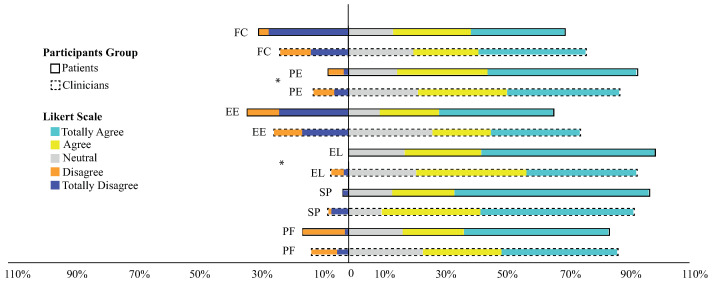
Perception between clinicians and patients. *Psychological factor* (PF), *Social perception* (SP), *Entertainment Level* (EL), *Effort’s Expectation* (EE), *Performance Expectation* (PE), and *Facility Conditions* (FC). (*) Significant differences between patients and clinicians.

The comparison between the participants (i.e., clinicians and patients) was also performed. In Figure 3, the perception regarding the clinicians and patients can be seen. The results showed that there is a positive perception towards the robot in both groups. As mentioned before, the negative perception can be primarily seen in the FC and EE constructs. These results represent that patients think that the robot’s usage can be complex (e.g., ease of use, understand, and follow up the robot instructions, among others) considering the EE construct. In order to observe if there exists a significant difference between clinicians and patients, a Mann-Whitney Test was applied to the data. Table 3 shows the p-values obtained through the test. As it can be seen in the table, the *Entertainment Level* and *Performance Expectancy* are different in both groups.

**Figure 4:**
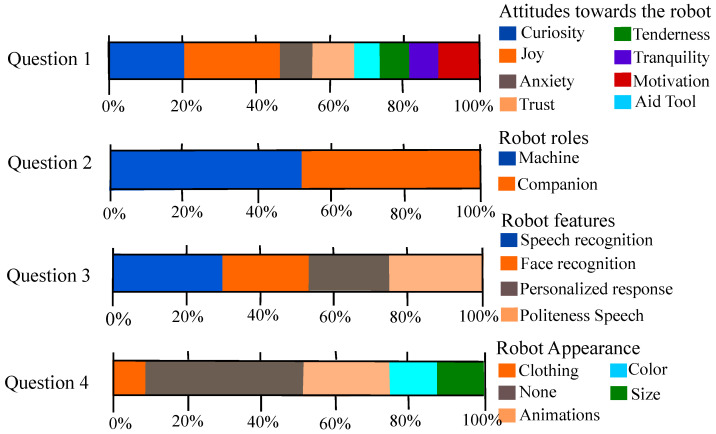
Results regarding the open questions of the UTAUT questionnaires.

Finally, the open questions were also analyzed. Figure 4 shows the frequency of the answers regarding essential aspects of a social robot role during the therapy. The attitudes and feelings towards the robot were analyzed through Question 1. As the figure shows, the two most common answers include that the robot produces *joy* and *curiosity* to the participants (21.31% and 26.22%), which shows a positive perception. On the contrary, some participants answer that the robot produces anxiety within them, which shows that a few participants have a negative perception of the robot. Question 2 focuses on evaluating of the perception of the robot by its role during the therapy. In this case, half of the participants see the robot as a *machine* and the other half as a *companion*. Question 3 evaluates the perception of the participants regarding the features developed within the robot. The participants can answer two or more options in this question, as Figure 4 elucidates that the patients preferred several features such as *Speech Recognition*, *Face Recognition*, *Peer Recognition*, and *Politeness Speech*.

Furthermore, the appearance of the robot was also introduced to the participants. Table 4, Question 4 shows that most of the participants would not change the physical appearance of the robot (43%). Other answers such as *Animations* and *Color* could be appreciated.

## 4. Discussion

In this paper, a survey to measure the participants’ perception and expectation towards a social robot is presented. A total of 88 participants answered the survey based on the UTAUT questionnaire. Different dimensions were evaluated through different constructs (e.g., *Physiological Factor*, *Social Perception*, *Performance expectations* among others). This questionnaire was applied to clinicians and patients involved in PR procedures based on Lokomat therapies in two different countries (Colombia and Spain).

Overall, a positive perception was seen in both groups of participants (i.e., clinicians and patients). The analysis of the information demonstrates that the population has a level of acceptance more than 60% in each UTAUT construct towards the social robot in PR with Lokomat. This result is very encouraging as the robot can be introduced initially in this scenario. Measuring the perception and acceptance in the first stage allows to have an initial perspective of the needs and expectations of the participants. Moreover, it is essential to show the participants the operating aspects of the technology (i.e., robustness and capabilities of the SAR system) within the videos.

On the other hand, negative perceptions towards the robot in some constructs can be seen. In the case of *Effort’s Expectation* and *Facility Conditions*, the participants ranked with a lower score in the Likert Scale (51.14% and FE, 43.63%). This result is interesting as it shows that the patients perceive that social robot usage can be complex. This perception is expected as the interaction with the robot is unknown for the users. In the literature, several studies [35,36] recommend performing an initial stage where the participants could interact with the technology and understand it in order to increase the acceptance of the robot in the time. For instance, the results regarding *Facility Conditions* which measure the capabilities of the robot to interact in other environments or ease the therapy showed to be more negative than other constructs. Analyzing the answers of clinicians and patients, both groups answered with a “1” to “2” score to the question: “I would like the robot to reduce the tasks that I have during the rehabilitation procedure”. This shows that clinicians do not want their tasks to be reduced by getting support from the robot. Showing that it is essential to clarify at the beginning of the study the purposes and limitations of the robot in the therapy. Furthermore, this negative perception increases in the patient group, which demonstrates that patients want to perform the rehabilitation procedure as the health-care staff establishes.

Concerning the comparison in both groups, it can be seen a difference between the perception of the *Entertainment Level* and *Performance Expectation* of clinicians and patients. Analyzing the results of the *Entertainment Level* construct, the clinicians ranked more questions with “Totally disagree” and “Disagree” than the patients, which show that patients perceive more the robot as an enjoyment tool. Most of the patients think that the therapy with the robot can be more amusing than conventional therapy. Furthermore, the *Performance Expectation* construct is different between clinicians and patients, showing that the expectation of the robot’s role during the therapy depends on the nature of the user during the therapy. For example, the patients answer more positively to the question that refers to the adherence (i.e., “The attendance can be more continuous when the robot takes part in the therapy”).

The open questions also represent the participant’s perception of the robot. As it was mentioned in the results; several dimensions were assessed in the questionnaire. In the literature, several studies have been demonstrated that the robot characteristics can influence the patient’s attitudes [35,37]. In the questionnaire, four key questions were implemented (attitudes/feelings towards the robot, robot role, robot features, and appearance) to evaluate the expectations and perception of the participants.

In the case of Open Question 1, most of the participants expressed feeling joy and curiosity which showed a positive result. This curiosity feeling can be related to the *Novelty Effect* produced when a new technology is introduced to the participants. It can also be seen that the negative feelings as anxiety are less presented throughout this question. In the case of the robot role perception, half of the participants see the robot as a machine and the other half as a companion. This can be due to the social presence of the robot not being perceived in the video. However, it does not represent a negative result, as commonly the robots are being seen as machines that aid the users.

Finally, for Open Question 3, most of the participants answer that the robot has to integrate speech recognition. This could be due to the user’s interest or have a natural interaction with the robot through speech [38]. The results regarding Open Question 4, showed that the participants would not change the appearance of the robot, which is very interesting. In this case, this result is expected, as in several studies it has been demonstrated that the adults/elderly users preferred humanoid-based appearance [39,40].

Within the study, it is worth highlighting the limitations; as it is an online questionnaire, it is not entirely guaranteed that the participants have answered the questionnaire adequately. On the other hand, the number between patients and clinicians is not homogeneous, and there are more clinicians than patients.

## 5. Conclusions

Physical disability has been increasing in recent years, which generates a greater number of people who need health services, such as physical and cognitive rehabilitation. Due to the increasing rates of disabilities, socially assistive robotics have been implemented in health care areas to support the activities of the physiotherapists and to provide better rehabilitation process to the patients.

In this context, this paper presents the perception and acceptance of a social robot in a PR scenario based on Lokomat therapies using a UTAUT questionnaire. Overall, a positive perception could be observed in the participants (>60% accept the social robot) who take part in the study, showing the potential of this tool within the support of the Lokomat therapy. Despite the benefits of the Lokomat, which is an assistive technology, the results showed that the need for social and cognitive support is also essential for the clinicians and the patients.

In the case of the *Efforts Expectation* and *Facility Conditions*, a negative perception can be seen. Most of the patients and clinicians think that robot usage can be complex, suggesting that an introduction phase is needed to implement the robot in the future. Between patients and clinicians, the perception differs in the *Entertainment Level* and *Performance Expectation*. This shows that the expectation varies according to the type of interaction performed by the participants. On the other hand, the open questions showed that participants have positive feelings over the robot and recommend interesting features that can improve communication and interaction in a posterior implementation.

For future work, a study that integrates the social robot in both clinics will be conducted. Considering the results of the perception assessed during this research, features, and requirements will be adjusted to the system. Furthermore, the limitations regarding the size, pathologies and socio-cultural profiles will be considered in future stages.

## Figures and Tables

**Table 1:** Participants Demographic Data.

	Clinicians (*n* = 77)	Patients (*n* = 11)
Age (years), mean (SD)	27.87, 9.43	33.63, 15.70
Gender, (n)	Female (58) Male (19)	Female (8) Male (8)
Profession, Patients diagnosis	Physiotheraphist (50) Therapist (8) Physiatrist (1) Other professions (18)	Cerebral Palsy

**Table 2:** UTAUT constructs definitions.

Construct	Definition
Psychological Factor	The degree to which an individual believes that the use of technology will generate trust and comfort during its implementation.
Social Perception	The degree to which an individual perceives that others value the use of the system.
Entertainment Level	The degree to which an individual considers that the technology used provides fun and entertainment when it is used.
Effort’s Expectation	The ease of use perceived by the user towards the system.
Performance Expectation	The degree to which an individual believes that the use of the system will help to obtain a benefit in his/her self-performance.
Facility Conditions	The degree to which an individual considers an organized and technical structure helps her or him adapt to the technology.

**Table 3:** Results of the constructs comparison between clinicians and patients ( Mann Withney Test).

Construct	Mean (Patients) ± SD	Mean (Clinicians) ± SD	*p*-Value
PF	4.02 ± 0.32	4.87 ± 0.13	0.16
SP	4.85 ± 0.09	4.76 ± 0.24	0.06
EL	4.98 ± 0.18	4.29 ± 0.21	p<0.01
EE	3.67 ± 0.52	2.98 ± 0.43	0.57
PE	3.99 ± 0.70	4.78 ± 0.56	p<0.01
FC	4.23 ± 0.25	3.85 ± 0.28	0.27

**Table 4:** Results regarding the open questions of the UTAUT questionnaires.

Questions	Selected Vocabulary	Frecuency
What does the robot inspire you?	Curiosity	13
	Joy	16
	Anxiety	5
	Trust	8
	Aid Tool	3
	Tenderness	5
	Tranquility	5
	Motivation	6
How do you perceive the robot?	Machine	46
	Companion	42
What kind of interactions or gestures should a robot have for a closer interaction?	Politeness Speech	17
	Speech Recognition	23
	Personalized Response	17
	Facial Recognition	17
What physical changes would it make to the robot?	Animations	15
	Clothing	7
	Color	10
	Size	11
	None	32

## Data Availability

The raw data supporting the conclusions of this article will be made available by the authors, without undue reservation.

## Appendix A

**Table A1:** Acceptance Questionnaire for Lokomat Therapy Users.

Construct	No.	Questions
PF	1	I am afraid of damaging the robot.
	2	I think that using the robot during the Lokomat sessions will be comfortable.
	3	Using the robot will generate stress.
	4	I think that the robot express emotions during the sessions will be uncomfortable.
	5	I think that the robot will increase the concentration during the therapy.
	6	I think that the robot speech could be weird.
	7	I think that using the robot will make me feel more secure.
	8	I think that the robot will give me confidence.
SP	1	I think that using the robot in the rehabilitation could be more interesting.
	2	I think that the interaction with the robot would be pleasant.
	3	I think that it is appropriate that the robot will use the data of the rehabilitation.
	4	Using the robot will give me satisfaction.
	5	I think that the robot’s instruction will be highly relevant.
EL	1	I think that the therapy could turn boring with the use of the robot.
	2	I think that I will enjoy more the therapy with the robot.
	3	I think that following-up with the robot’s instructions will be fun.
	4	I think that the robot company will make the therapy more enjoyable.
EE	1	Following the robot’s instructions would be difficult.
	2	I think that using the robot would improve Lokomat therapy.
	3	I think that I will be able to use the robot without external help.
	4	I think that use the robot will be easy.
	5	I think I will learn quickly how to use the robot.
PE	1	I think that the robot will be helpful during the rehabilitation process.
	2	The robot would help to decrease the fatigue during the therapies.
	3	I think that use the robot will make the therapies faster.
	4	I think that the presence of the robot is motivating.
	5	I think that the presence of the robot will affect over the engagement in the therapy.
	6	I think that using the robot is convenient for the rehabilitation procedure.
	7	I think that the use of the robot would become repetitive during the sessions.
	8	I think that the use of the robot will motivate the patients to perform better rehabilitation.
	9	I think that the use of the robot will make the session longer.
FC	1	I consider that I must have previous training before using the robot.
	2	I consider that the robot can be challenging to control.
	3	I consider that the robot could be adapted to any scenario.
	4	I consider that the robot could be used to assist patients with other pathologies.
	5	I would like the robot to reduce the workload I have during the rehabilitation procedure.
Open Questions	1	How you perceive the robot?.
	2	What does the robot inspire you?.
	3	What kind of interactions or gestures should a robot have for closer interaction?.
	4	Which physical changes would you make to the robot?.
